# Study on molecular mechanism of volatiles variation during *Bupleurum scorzonerifolium* root development based on metabolome and transcriptome analysis

**DOI:** 10.3389/fpls.2023.1159511

**Published:** 2023-03-24

**Authors:** Dan Yu, Wenxue Wang, Jinhai Huo, Yan Zhuang, Yiyang Chen, Xiaowei Du

**Affiliations:** ^1^ Key Laboratory of Chinese Materia Medica, Ministry of Education, Pharmaceutical College, Heilongjiang University of Chinese Medicine, Harbin, China; ^2^ Institute of Chinese Materia Medica, Heilongjiang Academy of Chinese Medicine Sciences, Harbin, China

**Keywords:** *Bupleurum scorzonerifolium* Willd., volatiles, fatty acids and their derivatives, metabolome, transcriptome

## Abstract

*Bupleurum scorzonerifolium* Willd. is a medicinal herb. Its root has a high content of volatile oil (BSVO), which shows a variety of biological activities. Currently, BSVO in the injectable form is used for treating fever in humans and livestock. The yield and quality of volatile oils depends on the developmental stages of plants. However, the changes in BSVO yield and quality during root development in *Bupleurum scorzonerifolium* and the underlying molecular regulatory mechanisms remain unclear. This knowledge gap is limiting the improvement in the quality of BSVO. In the present study, *B. scorzonerifolium* root was collected at germinative, vegetative, florescence, fruiting and defoliating stages. The yield of BSVO, metabolic profile of volatile components and transcriptome of root samples at various developmental stages were comprehensively determined and compared. BSVO continuously accumulated from the germinative to fruiting stages, and its level slightly decreased from the fruiting to defoliating stages. A total of 82 volatile components were detected from *B. scorzonerifolium* root, of which 22 volatiles were identified as differentially accumulated metabolites (DAMs) during the root development. Of these volatiles, fatty acids and their derivatives accounted for the largest proportion. The contents of most major volatiles were highest at the fruiting stage. A large number of differentially expressed genes (DEGs) were detected during *B. scorzonerifolium* root development, of which 65 DEGs encoded various enzymes and transcription factors regulating the biosynthesis of fatty acids and their derivatives. In further analysis, 42 DEGs were identified to be significantly correlated with DAMs, and these DEGs may be the key genes for the biosynthesis of volatiles. To the best of our knowledge, this is the first study to comprehensively report the changes in the composition and content of volatiles and underlying mechanism during *B. scorzonerifolium* root development. This study provided important reference for future studies to determine the harvest time of *B. scorzonerifolium* roots and improve the quality of BSVO.

## 1 Introduction

*Bupleurum scorzonerifolium* Willd. belongs to the genus *Bupleurum*, family Umbelliferae. It is widely distributed in China, as well as Russia (Siberia and the Far East), Mongolia, Korea and Japan. *B. scorzonerifolium* root has been recorded as an excellent herbal medicine as early as 2000 years ago in Shennong Bencao Jing (Shennong’s Classic of Materia Medica) in China. Nowadays, *B. scorzonerifolium* root has been recorded in the Chinese, British and European Pharmacopoeias as an official medicine, and it is used for the treatment of catching a cold, fever, inflammation and hepatitis (Pharmacopoeia, E.C.O.C, 2020; Sun et al., 2020). Up to now, phytochemical studies reported that *B. scorzonerifolium* root contained several kinds of natural constituents, such as triterpenoid saponins, volatile oils, flavonoids, polysaccharides, lignans and so on (Jiang et al., 2020; Qu et al., 2022). In China, *B. scorzonerifolium* is known as “Xiang Chaihu”, because it contains a high content of volatile oil giving it a strong odor. Notably, the volatile oil of *B. scorzonerifolium* root (BSVO) exhibits a variety of biological activities, such as antibacterial, antipyretic, analgesic, anti-inflammation, anti-depression, insecticide and so on (Li et al., 2007; Ashour and Wink, 2010; Li et al., 2022). Currently, injectable BSVO is used for treating fever in humans and livestock.

Changes in the chemical composition and yield of volatile oil are associated with plant developmental stage (Stefanini et al., 2006; Norouzi and Norouzi, 2018). In some volatile-oil-producing plants, the content of volatile oils increases during bud formation to full bloom stages, whereas in some plants, the accumulation of volatile oils significantly accompany the fruit ripening process (Sousa et al., 2021). It can be seen that the yield and quality of volatile oils continuously change during plant development. Thus, understanding the changes in yield and quality of BSVO during *B. scorzonerifolium* root development is helpful for determining the harvest time of *B. scorzonerifolium* root. Moreover, elucidating the underlying molecular mechanisms is useful for improving the quality of BSVO. However, relevant studies are unavailable.

In this work, the metabolic profile of volatiles in *B. scorzonerifolium* root was analyzed using gas chromatography-mass spectrometry (GC-MS), and it was found that most volatiles were fatty acids and their derivatives, such as fatty acids, fatty aldehydes, fatty alcohols and fatty esters, as well as terpenes and phenols in small amounts. Fatty acid metabolism pathway is a key pathway for the biosynthesis of fatty acids and their derivatives in plants. Here, this research presents the variation in the content and composition of volatiles and transcriptome in *B. scorzonerifolium* root during various developmental stages, which was comprehensive enough to analyze BSVO quality changes and related gene regulation mechanisms. This study can be used to propose a harvesting strategy for *B. scorzonerifolium* root and provides a theoretical basics for future research on the quality improvement of BSVO.

## 2 Material and methods

### 2.1 Plant materials

*B. scorzonerifolium* has been planted in the Huayuan Town, Daqing City, Heilongjiang Province, China, since last 2 years. The cultivated area is flat, humid and fertile. The root samples were collected at different developmental stages of *B. scorzonerifolium*, including germinative (BS_1, 28 April 2021), vegetative stage (BS_2, 11 June 2021), florescence (BS_3, 26 July 2021), fruiting stages (BS_4, 2 September 2021) and defoliating (BS_5, 23 October 2021) stages. Some roots were dried in shade for the determination of BSVO yield, and others were stored at -80°C for the metabolome and transcriptome analyses.

### 2.2 Analysis of BSVO yield

BSVO was extracted by steam distillation. Powder of *B. scorzonerifolium* root (100 g) were immersed in 1000 mL distilled water for 4 h in a round-bottom flask assembled with the volatile oil collection device. Subsequently, the extraction was performed by steam distillation for 4 h and was stopped when the yield of BSVO was no longer increasing. BSVO was collected, and dried with anhydrous sodium sulfate. Further, the oil phase was separated and weighed (m). Finally the yield of BSVO was calculated according to the formula: m/100×100 %. Samples from each developmental stage were subjected to three replicates in parallel.

### 2.3 Analysis of metabolic profile of the volatiles

Frozen roots of *B. scorzonerifolium* (1 g) were ground into powder in liquid nitrogen. To the powder, 1 mL of prechilled petroleum ether (Merck, Darmstadt, Germany) and 2 µL of internal standard (cyclohexane, Aladdin, Shanghai, China) was added, and extraction was conducted for 24 h at 4 °C. The extract was centrifuged at 12,000 rpm in a refrigerated centrifuge (Sigma, Jiangsu, China) for 10 min. The supernatant was filtered with a microfiltration film before GC-MS analysis.

Volatiles were analyzed using GC-MS on a Perkin-Elmer Clarus SQ 8T (Massachusetts, USA). An Elite-5MS capillary column (60 m×0.32 mm×0.25 μm, Perkin-Elmer, Massachusetts, USA) was used to separate volatiles. Helium was used as a carrier gas at a constant flow rate of 1 mL/min. 1 µL of the sample was injected in a split mode (a ratio of 10:1). The initial oven temperature was programmed from 80°C (isothermal for 5 min), with an increase of 6°C/min to 165 °C, maintaining for 5 min, then 6°C/min to 220°C, ending with a 5 min isothermal at 220°C. An electron ionization system was taken at 70 eV, and the ion-source temperature was 250°C. The data were recorded in scan mode of m/z 40-450. The solvent delay was 0 to 5 min, and the total running time was 39.67 min.

The volatiles were identified and quantified by comparing MS fragmentation patterns with the NIST 14 library. The content of each compound was calculated by comparing its peak area with that of the internal standard. The processed data was multivariate-analyzed by SIMCA-P^+^ 14.1 (Umetrics). The differentially accumulated metabolites (DAMs) in ten groups (BS_1 vs BS_2, BS_1 vs BS_3, BS_1 vs BS_4, BS_1 vs BS_5, BS_2 vs BS_3, BS_2 vs BS_4, BS_2 vs BS_5, BS_3 vs BS_4, BS_3 vs BS_5 and BS_4 vs BS_5) were screened according to the following criteria: P value ≤ 0.05; variable importance in project (VIP) ≥ 1.

### 2.4 RNA isolation and transcriptome sequencing

*B. scorzonerifolium* root samples collected at five different developmental stages were prepared for transcriptome sequencing. Samples from each developmental stage were subjected to three replicates in parallel. Total RNA was extracted from the frozen root tissue using TRIzol^®^ Reagent (Invitrogen, CA, USA). The quality and quantity of the total RNA were checked using 2100 Bioanalyser (Agilent, CA, USA) and ND-2000 NanoDrop (Thermo, DE, USA). Subsequently, messenger RNA (mRNA) was purified from total RNA using TruSeqTM RNA sample preparation Kit (Illumina, CA, USA), and was used as a template to synthesize double-stranded cDNA using a SuperScript double-stranded cDNA synthesis kit (Invitrogen, CA, USA) with random hexamer primers (Illumina, CA, USA). Then cDNAs were subjected to end-repair, adapter ligation, PCR amplification and purification to create a cDNA library. Finally, the constructed library was sequenced on Hiseq xten/NovaSeq 6000 sequencer platform (Illumina, CA, USA) for 150-bp paired-end reads. All raw data in the study were deposited into NCBI (BioProject accession code PRJNA916499).

### 2.5 *De novo* assembly and annotation

The raw reads were filtered by SeqPrep (https://github.com/jstjohn/SeqPrep) and Sickle (https://github.com/najoshi/sickle) with default parameters. The clean data obtained by filtering were *de novo* assembled with Trinity (http://trinityrnaseq.sourceforge.net/) (Grabherr et al., 2011). Based on the National Center for Biotechnology Information (NCBI) non-redundant protein database (NR), Gene Ontology (GO), Non-supervised Orthologous Groups (eggNOG), protein families (Pfam), reviewed protein sequence database (Swiss-Prot), and Kyoto Encyclopedia of Genes and Genomes (KEGG) Ontology (KO) databases, all assembled transcripts were identified by selecting the given sequence with the highest similarity using BLASTX with an E-value cutoff of 10^−5^. The databases of BLAST2GO (http://www.blast2go.com/b2ghome) (Conesa et al., 2005) and KEGG (http://www.genome.jp/kegg/) (Kanehisa and Goto, 2000) were used for functional annotation and metabolic pathway analysis of the assembled transcripts.

### 2.6 Differentially expressed genes and functional enrichment

Transcript abundance was normalized to Fragments Per Kilobase of transcript per Million fragments mapped (FPKM) using RSEM (http://deweylab.biostat.wisc.edu/rsem/) (Dewey and Li, 2011). The significant differentially expressed genes (DEGs) in the ten groups (BS_1 vs BS_2, BS_1 vs BS_3, BS_1 vs BS_4, BS_1 vs BS_5, BS_2 vs BS_3, BS_2 vs BS_4, BS_2 vs BS_5, BS_3 vs BS_4, BS_3 vs BS_5 and BS_4 vs BS_5) were identified using the DESeq2 (Love et al., 2014) with |log2FC|>1 and Q value ≤ 0.05. Goatools (https://github.com/tanghaibao/Goatools) and KOBAS (http://kobas.cbi.pku.edu.cn/home.do) were used for GO and KEGG functional-enrichment analyses of DEGs based on the principle of Bonferroni-corrected P-value ≤ 0.05 compared with the full-transcriptome background (Chen et al., 2011).

### 2.7 Validation of transcripts using qRT-PCR

The reliability of RNA-seq data was validated using qRT-PCR on the AriaMx Real-Time PCR System (Agilent, CA, USA). The specific primers of transcripts and housekeeping gene (GAPDH) are listed in **Table S1** and were designed by Sangon Biotech (Sangon, Shanghai, China) and Takara Bio Inc. (Takara, Otsu, Japan) using Primer 5 in the laboratory. First-strand cDNA was synthesized using PrimeScript™ RT Reagent Kit (Takara, Otsu, Japan). The reaction (20 μL) program was as follows: 95°C for 30 s, followed by 40 cycles of 95 °C for 5 s and 65 °C for 30 s. Three technical replicates were included for each sample, and the relative expression levels for each gene were calculated using 2^−△△Ct^ method.

### 2.8 Correlation analysis of metabolome and transcriptome profiles of volatiles

The correlation analysis between DAMs and DEGs involved in the fatty acid metabolism pathway was performed using the Pearson's correlation analysis (SPSS 18.0). Log_2_ conversion of data was performed uniformly before the analysis. Correlations with a coefficient of R^2^ > 0.8 and P value ≤ 0.05 were selected. Cytoscape software (version 3.7.2) was used to visualize the network.

## 3 Results

### 3.1 The yield of BSVO at five developmental stages of *B. scorzonerifolium*

**Figure 1:**
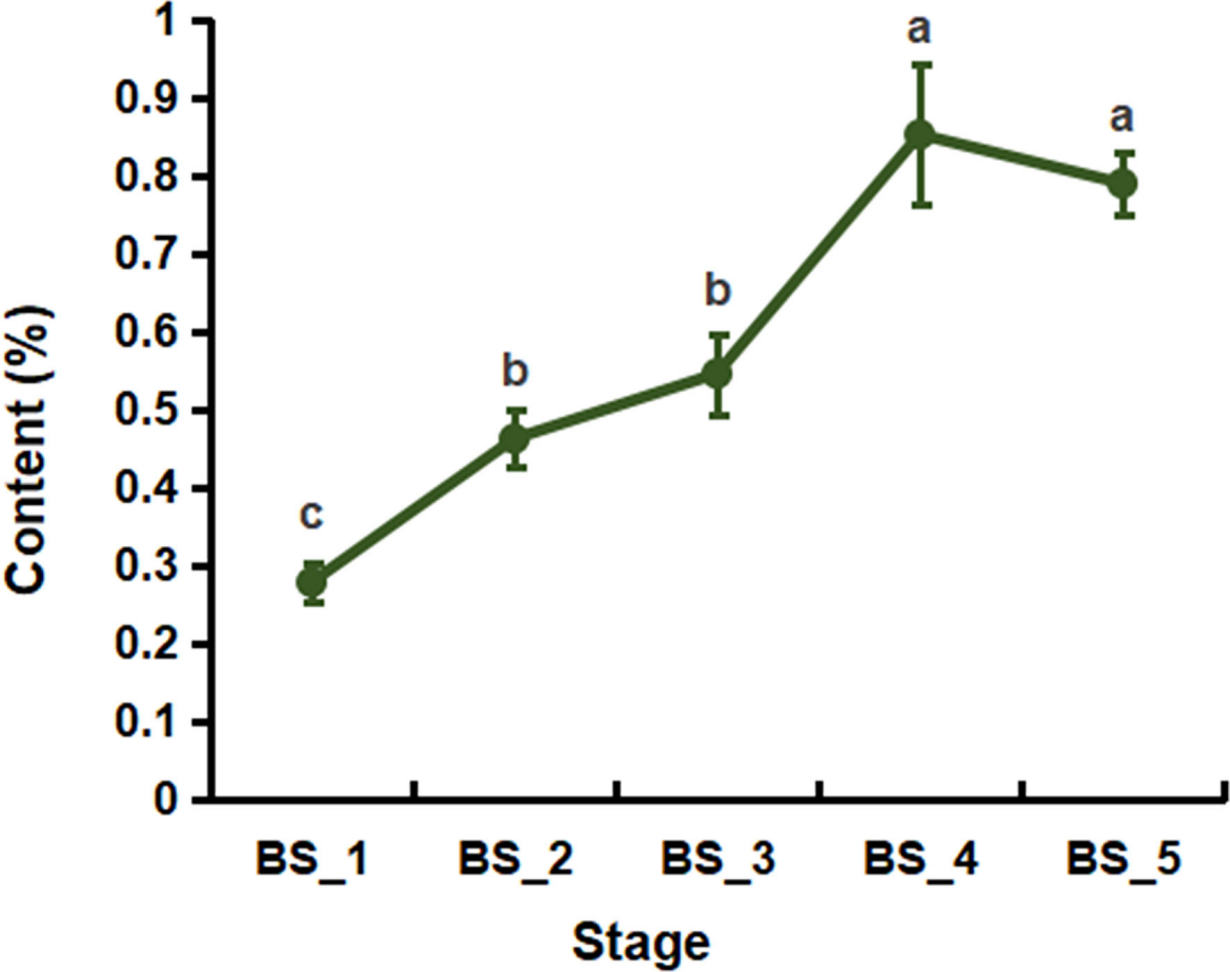
The yield of volatile oil from *Bupleurum scorzonerifolium* root at five developmental stages. Samples labeled BS_1, BS_2, BS_3, BS_4, and BS_5 represent the samples of *B. scorzonerifolium* root collected at germinative, vegetative, florescence, fruiting and defoliating stages, respectively. Different letters (a, b, c) indicate statistical significant differences.

As shown in **Figure 1**, from the BS_1 to BS_4 stages, the yield of BSVO continuously increased, reaching up to 0.86 % at the BS_4 stage. From the BS_4 to BS_5 stages, the yield of BSVO decreased slightly but without any significant difference (*P* > 0.05). The results showed that BSVO accumulated continuously during the root development and reached the peak at the fruiting stage.

### 3.2 Metabolic profiling of the volatiles

**Figure 2:**
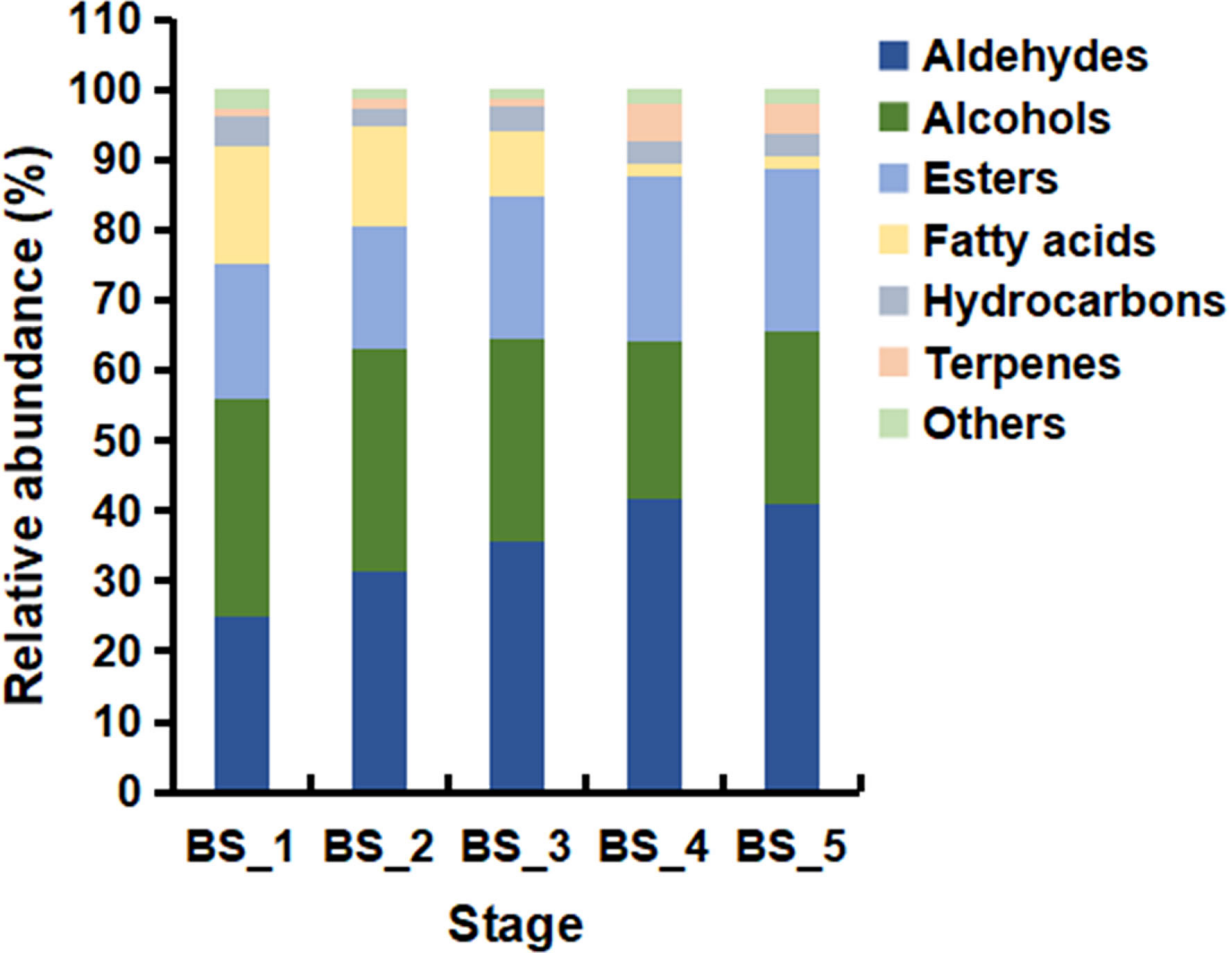
Relative abundance of different classes of volatiles in *Bupleurum scorzonerifolium* root at five developmental stages. Samples labeled BS_1, BS_2, BS_3, BS_4, and BS_5 are the samples of *B. scorzonerifolium* root collected at germinative, vegetative, florescence, fruiting and defoliating stages, respectively.

Based on GC-MS and NIST14 database, the volatile metabolites during the root development were studied. A total of 82 volatiles were identified, including 27 terpenes, 17 aldehydes, 10 alcohols, 10 esters, 7 hydrocarbons, 6 fatty acids, and 5 other compounds (**Table S2**). In total, 33, 52, 50, 55 and 48 volatiles were detected in the BS_1 to BS_5 stages, respectively, and a total of 23 volatiles were common in all samples. As shown in **Figure 2**, aldehydes, alcohols, and esters were abundant at the five developmental stages. Differently, fatty acids were higher at the BS_1, BS_2 and BS_3 stages and less at the BS_4 and BS_5 stages. In addition, terpenes were maintained at low levels during the root development with the highest content at the fruiting stage. The levels of hydrocarbons and other compounds were even less.

**Figure 3:**
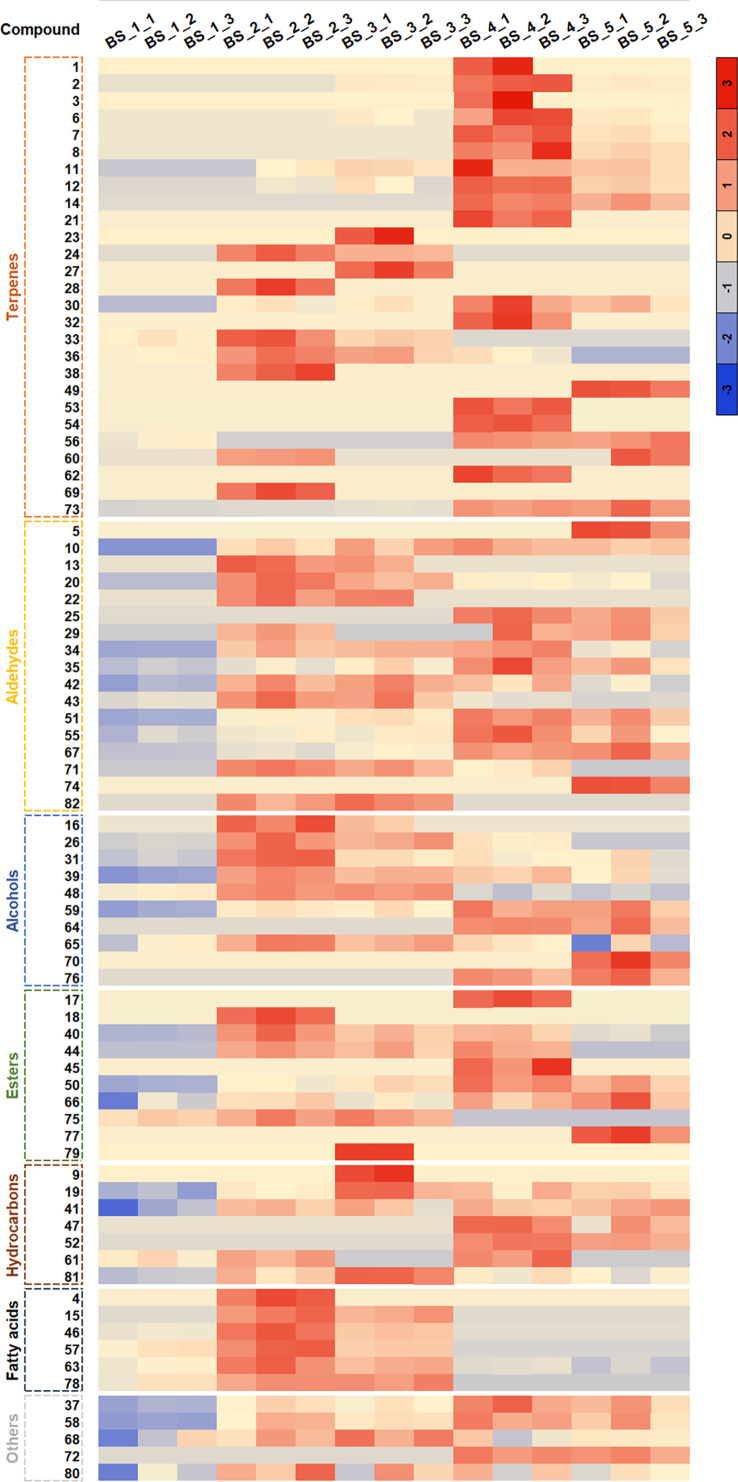
Cluster diagram of volatile metabolites in *Bupleurum scorzonerifolium* root at five developmental stages. The abscissa represents the sample name, and the ordinate represents the corresponding compound name. The color represents the value of the correlation coefficient. Samples labeled BS_1, BS_2, BS_3, BS_4, and BS_5 are the samples of *B. scorzonerifolium* root collected at germinative, vegetative, florescence, fruiting and defoliating stages, respectively.

As shown in **Figure 3**, the content of volatiles changed significantly during the root development. Among the 82 volatiles, dodecanal was the dominant compound, accounting for 22.00 %-35.32 % of the total volatiles at the five developmental stages, followed by decyl acetate (13.93 %-21.75 %), 1-dodecanol (12.80 %-17.78 %) and 1-decanol (5.00 %-11.42 %). Furthermore, the contents of dodecanal, decyl acetate and 1-dodecanol were the highest at the BS_4 stage, and that of 1-decanol was the highest at the BS_2 stage. In addition, dodecanoic acid (6.61 %-14.08 %) mainly accumulated at the BS_1, BS_2 and BS_3 stages, with the highest content at the BS_2 stage (**Table S2**).

### 3.3 DAMs analysis

**Figure 4:**
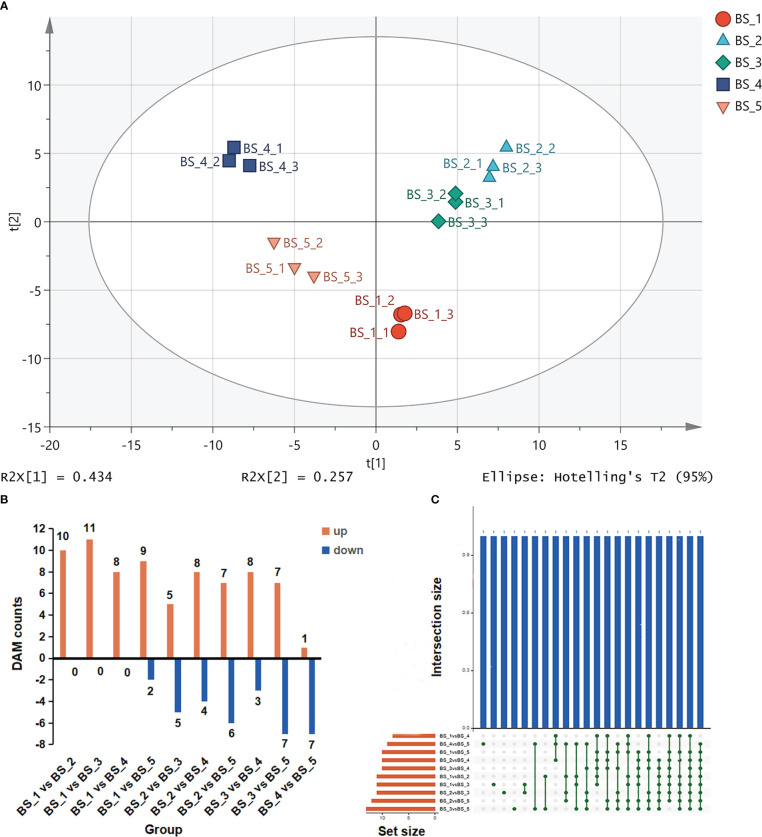
Differentially accumulated metabolites (DAMs) during *Bupleurum scorzonerifolium* root development. **(A)** Principal component analysis of the fifteen samples based on the volatile metabolic profiles. **(B)** The numbers of DAMs in different compared groups. **(C)** Venn diagram representing the DAMs in different compared groups. Samples labeled BS_1, BS_2, BS_3, BS_4, and BS_5 are the samples of *B. scorzonerifolium* root collected at germinative, vegetative, florescence, fruiting and defoliating stages, respectively.

Principal component analysis (PCA) results showed a trend of metabolome separation among the five groups (**Figure 4A**), indicating that there were differences in the volatiles in the root of *B. scorzonerifolium* collected at the different developmental stages.

A total of 22 compounds were identified as DAMs using OPLS−DA model (**Figures 4B, C** and **Table S3**). Most the of DAMs were fatty aldehydes (decanal, dodecanal, tetradecanal and undecanal), fatty acids (dodecanoic acid, decanoic acid, hexadecanoic acid and undecanoic acid), and terpenes [α-pinene, (Z, Z)- α-farnesene, β-longipinene and bicyclo[3.1.1]heptane, 6,6-dimethyl-2-methylene-, (1S)], this was followed by fatty alcohols (1-decanol, 1-dodecanol and 1-undecanol), esters (decyl acetate, dibutyl phthalate and isobornyl acetate), hydrocarbons and ethers. Among these DAMs, the levels of fatty aldehydes, fatty alcohols and fatty esters changed drastically during the root development. Compared with the BS_1 stage, the contents of dodecanal, decyl acetate, 1-decanol, 1-dodecanol, dodecanoic acid, decanal, 1-undecanol, decanoic acid and undecanal were significantly increased at the BS_2 stage. The contents of dodecanal, decyl acetate and tetradecanal were increased, whereas those of dodecanoic acid, 1-decanol, decanoic acid and undecanoic acid were decreased at the BS_3 stage compared with those at the BS_2 stage. The contents of dodecanal, decyl acetate, 1-dodecanol, 1-undecanol and tetradecanal were considerably increased, whereas those of dodecanoic acid and decanoic acid were decreased at the BS_4 stage compared with those at the BS_3 stage. Compared with the BS_4 stage, the contents of decanal, dodecanoic acid and undecanal at the BS_5 stage were decreased. The KEGG database was used to annotate these DAMs, which were mainly involved in the fatty acid metabolism pathway.

### 3.4 Quality assessment of sequencing data

Approximately 40.62-59.02 million raw reads were obtained from the roots of *B. scorzonerifolium* at various developmental stages, respectively. After a filtration of the raw data, 40.42-58.73 million clean reads remained, and the amount of clean data from 15 samples reached 103.58 GB. The percentage of Q20 and Q30 bases was more than 97.45 % and 92.85 %, respectively. The percentage of G and C bases among the total bases was 42.80 %-43.38 % (**Table 1**). A total of 173,834 unigene sequences with N50 lengths of 1,863 bp were obtained by assembling the high quality sequences. The assembled unigenes were annotated for gene functions in six databases (NR, Pfam, eggNOG, Swiss-Prot, KEGG and GO), and a total of 52,028 unigenes (60.12 % of all unigenes) were annotated in at least one database (**Table S4**).

**Table 1:** Summary of transcriptome data generated in *Bupleurum scorzonerifolium* root.

Sample	Raw Reads	Clean Reads	Clean bases	Error rate (%)	Q20 (%)	Q30 (%)	GC content (%)	Mapped ratio
BS_1_1	45,042,982	44,761,032	6.65G	0.02	98.36	94.88	42.93	35,765,190 (79.90%)
BS_1_2	44,820,002	44,528,958	6.61G	0.02	98.36	94.90	42.99	35,025,494 (78.66%)
BS_1_3	40,618,862	40,421,076	6.02G	0.02	98.33	94.73	42.99	31,935,528 (79.10%)
BS_2_1	53,913,542	53,667,352	7.97G	0.02	98.32	94.74	43.13	42,376,986 (78.96%)
BS_2_2	46,918,964	46,683,550	6.93G	0.02	98.30	94.70	43.38	33,582,552 (78.36%)
BS_2_3	42,848,826	42,620,602	6.32G	0.02	98.33	94.79	42.99	33,747,454 (79.18%)
BS_3_1	42,574,788	42,305,756	6.31G	0.02	98.16	94.29	42.85	32,912,980 (77.80%)
BS_3_2	46,416,914	46,078,306	6.84G	0.02	98.25	94.56	42.80	37,271,246 (80.89%)
BS_3_3	59,015,392	58,733,332	8.72G	0.02	98.38	94.82	43.31	48,053,182 (81.82%)
BS_4_1	51,369,948	50,899,980	7.42G	0.03	97.62	93.20	43.25	42,038,822 (82.59%)
BS_4_2	47,844,766	47,423,454	6.94G	0.03	97.58	93.18	43.32	38,893,470 (82.01%)
BS_4_3	47,746,606	47,298,426	6.91G	0.03	97.54	93.00	43.34	38,843,122 (82.12%)
BS_5_1	47,976,938	47,529,680	6.94G	0.03	97.45	92.85	43.33	38,931,844 (81.91%)
BS_5_2	45,769,254	45,377,958	6.64G	0.03	97.57	93.13	43.32	36,423,672 (80.27%)
BS_5_3	43,904,828	43,535,222	6.37G	0.03	97.63	93.29	43.37	35,243,412 (80.95%)

The expression of total transcripts was subjected to PCA analysis. The three biological replicates of each developmental stage clustered together well, and the samples of five developmental stages obviously separated. This grouping indicated that the RNA-Seq data varied among the five developmental stages. The result was shown in **Figure S1**.

### 3.5 Enrichment of DEGs

**Figure 5:**
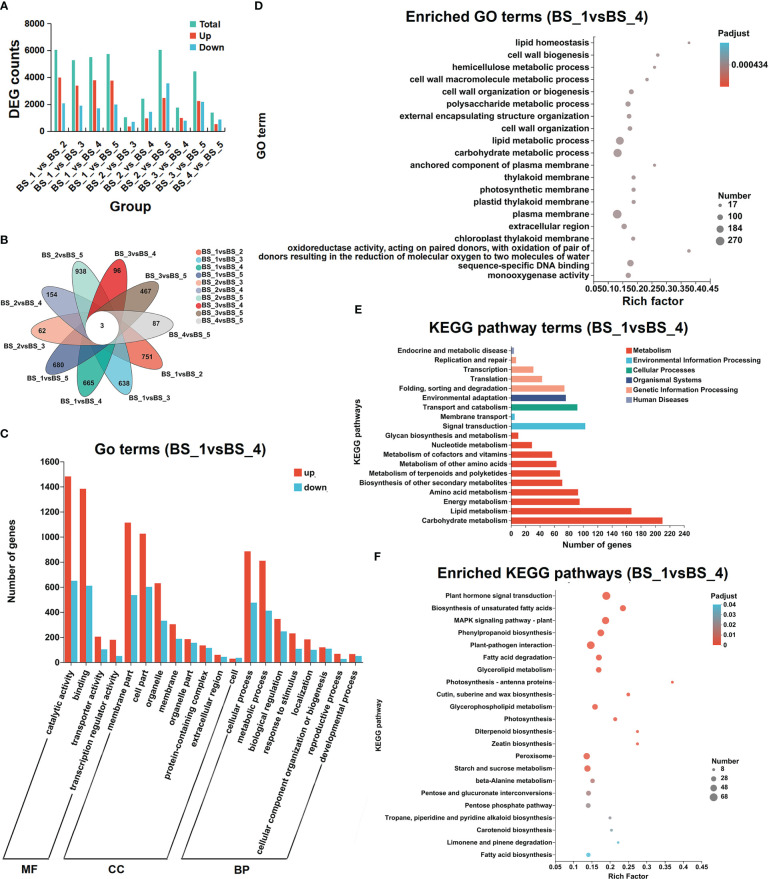
Differentially expressed transcripts during *Bupleurum scorzonerifolium* root development. **(A)** Total numbers of differentially expressed genes (DEGs), upregulated genes, and downregulated genes in different compared groups. **(B)** Venn diagram of the DEGs in different compared groups. **(C)** GO annotation analysis of the upregulated genes and downregulated genes in BS_1 vs BS_4 group. **(D)** GO enriched analysis of DEGs in BS_1 vs BS_4 group. **(E)** KEGG annotation analysis of DEGs in BS_1 vs BS_4 group. **(F)** KEGG enriched analysis of DEGs in BS_1 vs BS_4 group. Samples labeled BS_1, BS_2, BS_3, BS_4, and BS_5 are the samples of *B. scorzonerifolium* root collected at germinative, vegetative, florescence, fruiting and defoliating stages, respectively.

Based on the DEG-Seq analysis, an overview of the DEGs in ten groups was obtained. The numbers of DEGs in each group are represented in **Figure 5A** and **Table S5**. The most DEGs were found in the BS_1 vs BS_2 group (including 3,984 upregulated and 2,068 downregulated transcripts), followed by BS_2 vs BS_5 group (2,477 upregulated and 3,573 downregulated transcripts), BS_1 vs BS_5 group (3,759 upregulated and 1,976 downregulated transcripts), BS_1 vs BS_4 group (3,784 upregulated and 1,711 downregulated transcripts), and BS_1 vs BS_3 group (3,381 upregulated and 1,903 downregulated transcripts). Fewer DEGs were present in the BS_2 vs BS_3, BS_3 vs BS_4, and BS_4 vs BS_5 groups. The Venn diagram (**Figure 5B**) indicated that the DEGs commonly existed in the above ten groups. These DEGs might be relevant to the formation of the volatiles.

To further explore the possible functions of DEGs, the GO enrichment analysis was performed. The DEGs of ten groups were all classified into three major functional categories, including cellular components (CC), molecular functions (MF), and biological processes (BP). The dominant enriched GO terms for the ten groups were catalytic activity (GO:0003824) and binding (GO:0005488) in MF, cell part (GO:0044464) and membrane part (GO:0044425) in CC, and cellular process (GO:0009987) and metabolic process (GO:0008152) in BP (**Figures 5C**, **S2**). In GO enrichment analysis, the DEGs were classified according to the GO annotation results (**Figures 5D**, **S3**). In particular, in the BS_1 vs BS_4 group, the DEGs were significantly enriched in lipid homeostasis and lipid metabolic process terms.

The KEGG pathway annotation and enrichment analysis was conducted to determine the metabolic pathways and functions of DEGs. Most of the DEGs were concentrated in metabolism, particularly its subcategories such us carbohydrate metabolism and lipid metabolism (**Figures 5E**, **S4**). Based on the selected threshold value of P value corrected <0.5, these DEGs enriched in fatty acid biosynthesis pathway (map00061), biosynthesis of unsaturated fatty acid (map01040), fatty acid degradation pathway (map00071), and alpha-linolenic acid metabolism (map00592) (**Figures 5F**, **S5**). These results indicated that the genetic manifestations of these pathways were active during the root development, providing a clear direction for elucidating the molecular mechanisms underlying the accumulation and changes in the volatiles.

### 3.6 Metabolic pathway analysis of candidate genes involved in the biosynthesis of volatiles

**Figure 6:**
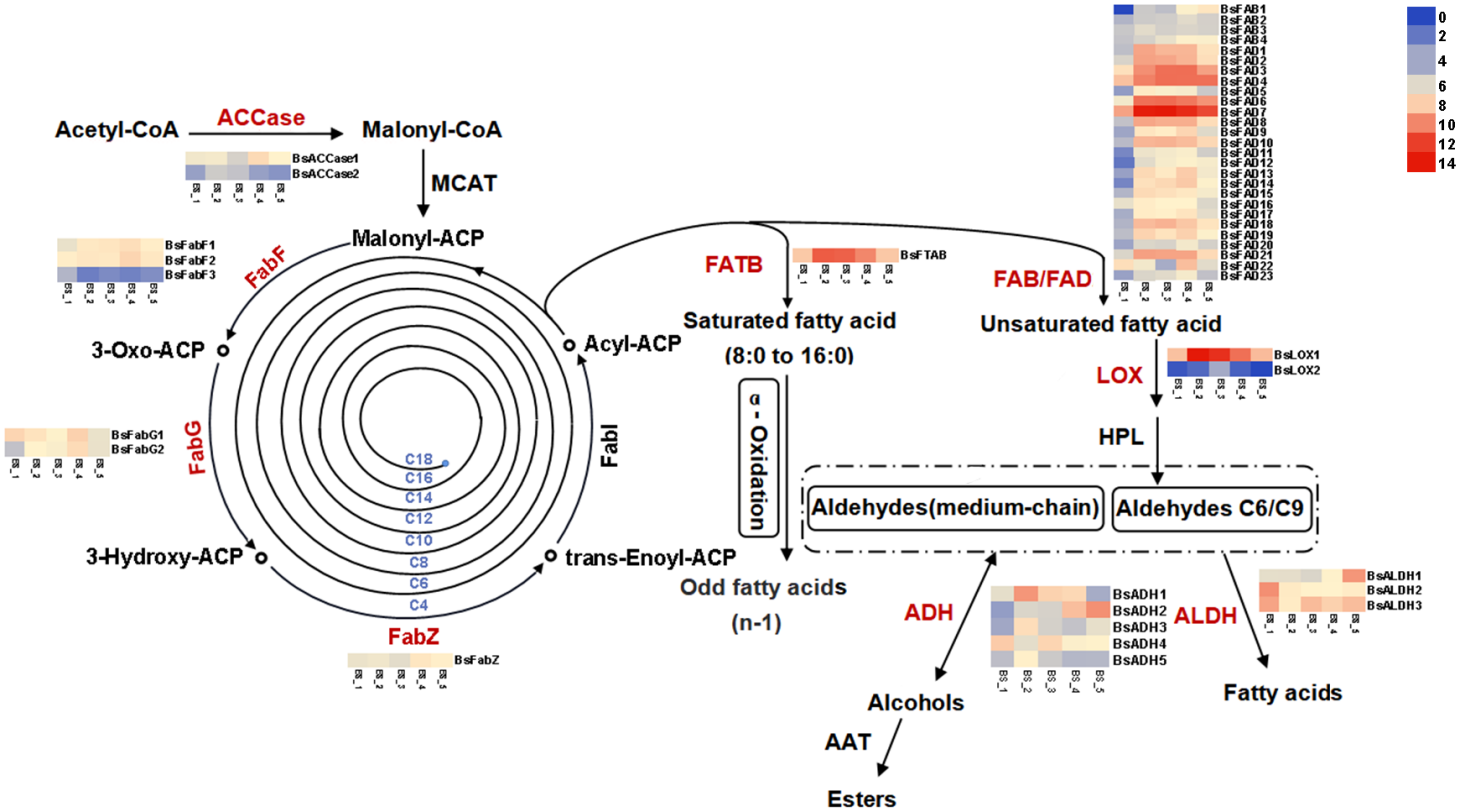
Pathways and genes involved in biosynthesis of fatty acids and their derivatives in *Bupleurum scorzonerifolium* root. Acetyl-CoA carboxylase (ACCase), 3-Oxoacyl-ACP synthase (FabF), 3-Oxoacyl-ACP reductase (FabG), 3-Hydroxyacyl-ACP dehydratase (FabZ), Fatty acyl-ACP thioesterase B (FATB), Stearoyl-ACP desaturase (FAB), Fatty acid desaturase (FAD), Lipoxygenase (LOX), Hydroperoxide lyase (HPL), Aldehyde dehydrogenase (ALDH), Alcohol dehydrogenase (ADH), Alcohol acyltransferase (AAT).

The fatty acid metabolic pathway is the key pathway for the generation of fatty acids and their derivatives. In the present study, the DEGs included some genes encoding synthases involved in the fatty acid metabolism pathway (**Figure 6** and **Table S6**). Based on the annotation information of the transcriptome, a total of 46 DEGs with FPKM values greater than 1 were involved in the fatty acid metabolism pathway, and they encoded in 10 enzymes, namely acetyl-CoA carboxylase (ACCase), 3-oxoacyl-ACP synthase (FabF), 3-oxoacyl-ACP reductase (FabG), 3-hydroxyacyl-ACP dehydratase (FabZ), fatty acyl-ACP thioesterase B (FATB), stearoyl-ACP desaturase (FAB), fatty acid desaturase (FAD), lipoxygenase (LOX), aldehyde dehydrogenase (ALDH) and alcohol dehydrogenase (ADH).

Malonyl-CoA forms from acetyl-CoA catalyzed by ACCase (Tomokazu et al., 1996). Two genes encoding ACCase, *BsACCase1* and *BsACCase2*, were highly expressed at the BS_4 and BS_2 stages, respectively. FabF, FabG and FabZ were responsible for converting malonyl-ACP into a long-chain acyl-ACP (Dehesh et al., 2001; Brown et al., 2006; Zhang et al., 2013). Two genes (*BsFabF1* and *BsFabF2*) encoding FabF, two genes (*BsFabG1* and *BsFabG2*) encoding FabG, and a gene (*BsFabZ*) encoding FabZ exhibited the highest expression at the BS_4 stage, and the gene (*BsFabF3*) encoding FabF was highly expressed at the BS_1 stage. Acyl-ACP either generates saturated fatty acid *via* FATB regulation (Jones et al., 1995; Liu et al., 2013) or is desaturated to form unsaturated fatty acid, which is catalyzed by FAB and FAD, respectively (Li et al., 2021). The gene (*BsFTAB*) encoding FATB exhibited the highest expression at the BS_2 stage, and multiple genes encoding FAB (from *BsFAB1* to *BsFAB4*) and FAD (from *BsFAD1* to *BsFAD23*) were highly expressed at the BS_2, BS_3 and BS_4 stages. LOX was responsible for the production of C6 or C9 aldehyde (Schwab et al., 2008). Two genes encoding LOX, *BsLOX1* and *BsLOX2*, were identified, and they highly expressed at the BS_2, BS_3 and BS_4 stages. Additionally, ALDH can oxidize fatty aldehyde to produce carboxylic acid (Islam and Ghosh, 2022). In this study, three genes (*BsALDH1*, *BsALDH2* and *BsALDH3*) encoding ALDH were annotated. However, the trends of their up- or down-regulation during the root development were not consistent. ADH is a key enzyme in reducing fatty aldehyde to fatty alcohol (Dudareva et al., 2013). A total of five genes encoding ADH were identified. *BsADH1*, *BsADH3* and *BsADH5* were highly expressed at the BS_2 stage, *BsADH2* expression continuously increased, and that of *BsADH4* constantly changed during the root development. Alcohol acyltransferase (AAT) is responsible for the conversion of fatty alcohol to ester (Goff and Klee, 2006). Two genes encoding AAT were annotated. However, they showed no significant difference at the five developmental stages. Overall, most of these genes were highly expressed at the BS_2, BS_3 and BS_4 stages, and lowly expressed at the BS_1 and BS_5 stages. The over-expression of genes promoted the accumulation of fatty acids and their derivatives in *B. scorzonerifolium* root, particularly at the vegetative, florescence and fruiting stages. These results were consistent with the metabolomic findings.

### 3.7 Identification of transcription factors

**Figure 7:**
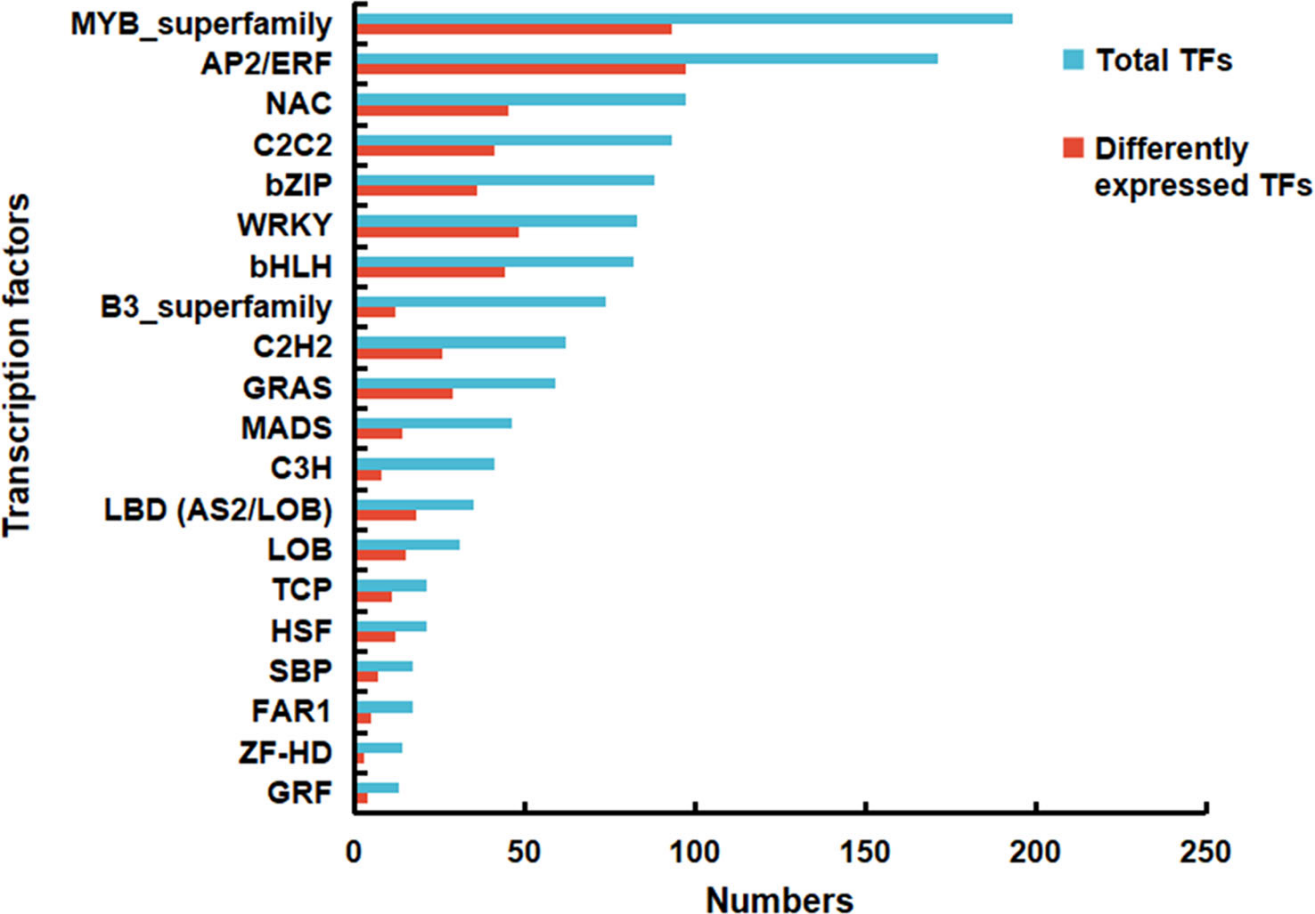
Total genes and differentially expressed genes (DEGs) occupied a proportion of the transcription factors in the de novo assembled transcriptome of *Bupleurum scorzonerifolium* root.

Transcription factors (TFs) are the key regulators that play crucial roles in the signal transduction pathway. In the current study, a total of 1336 TFs were detected, which were classified into 33 families. Among these families, MYB and AP2/ERF represented the dominant categories comprising 193 (14.45 %) and 171 (12.80 %) transcripts, respectively. Similarly, among the 584 DEGs annotated as TFs, AP2/ERF and MYB family were more abundant, accounting for 97 (16.64 %) and 93 (15.95 %) transcripts, respectively. The top 20 TF families are displayed in **Figure 7** and **Table S7**. It was worth noting that the TFs of MYB (MYB73 and MYB123), WRKY (WRKY6 and WRKY43), TCP (TCP4 and TCP20), GRAS (DELLA), C2C2 (dof4.6 and dof11) and AP2/ERF (RAP2.3) are reported to regulate the biosynthesis of fatty acids and their derivatives (Yang et al., 2022). Most of them were highly expressed in the vegetative, florescence and fruiting stages (**Table S8**). These findings suggested that the identified TFs were valuable regulator candidates for the accumulation of BSVO in *B. scorzonerifolium* root.

### 3.8 Correlation analysis between DAMs and DEGs

**Figure 8:**
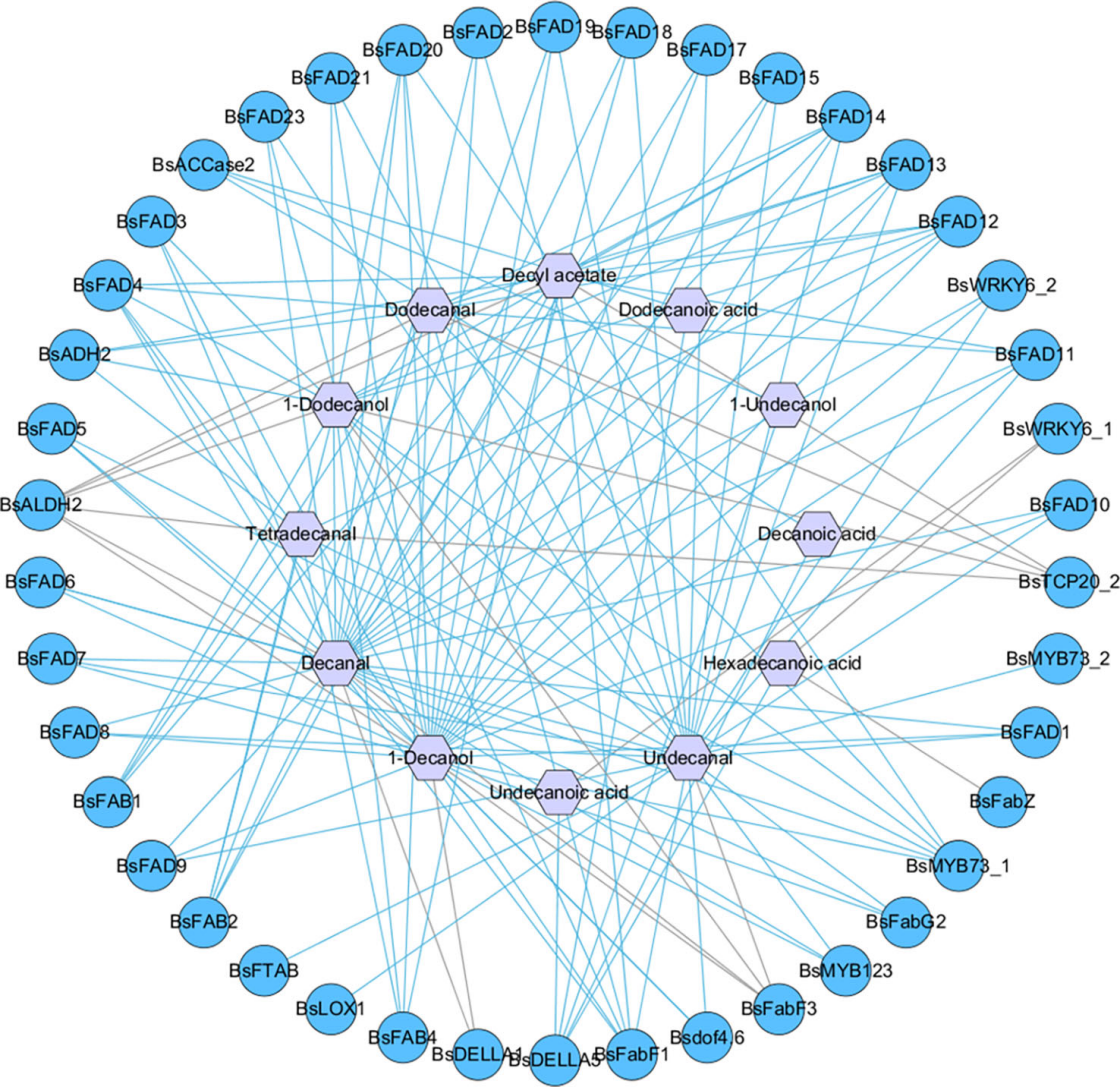
Gene-metabolite Pearson correlation network between differentially expressed genes (DEGs) and differentially accumulated metabolites (DAMs). Gene-metabolite pairs were connected by edges; light blue and grey lines represent positive and negative correlation respectively; circle and hexagon represent DEGs and DAMs, respectively.

To determine the key genes regulating the biosynthesis of fatty acids and their derivatives, correlation analysis between 12 DAMs and 65 DEGs involved fatty acid metabolism pathway was performed. The results showed that 42 DEGs were positively or negatively correlated with DAMs. *BsFabF1*, *BsFAD4*, *BsFAD12*, *BsFAD13*, *BsFAD14*, *BsALDH2*, *BsMYB73_1*, *BsFAB2*, *BsFAD11*, *BsFAD20* and *BsDELLA5* were highly correlated with decanal, 1-decanol, undecanal, dodecanal, 1-dodecanol and decyl acetate, indicating that these genes had a greater impact on the biosynthesis of these volatiles. The results are shown in **Figure 8** and **Table S9**.

### 3.9 Validation of DEG profiles using qRT-PCR

**Figure 9:**
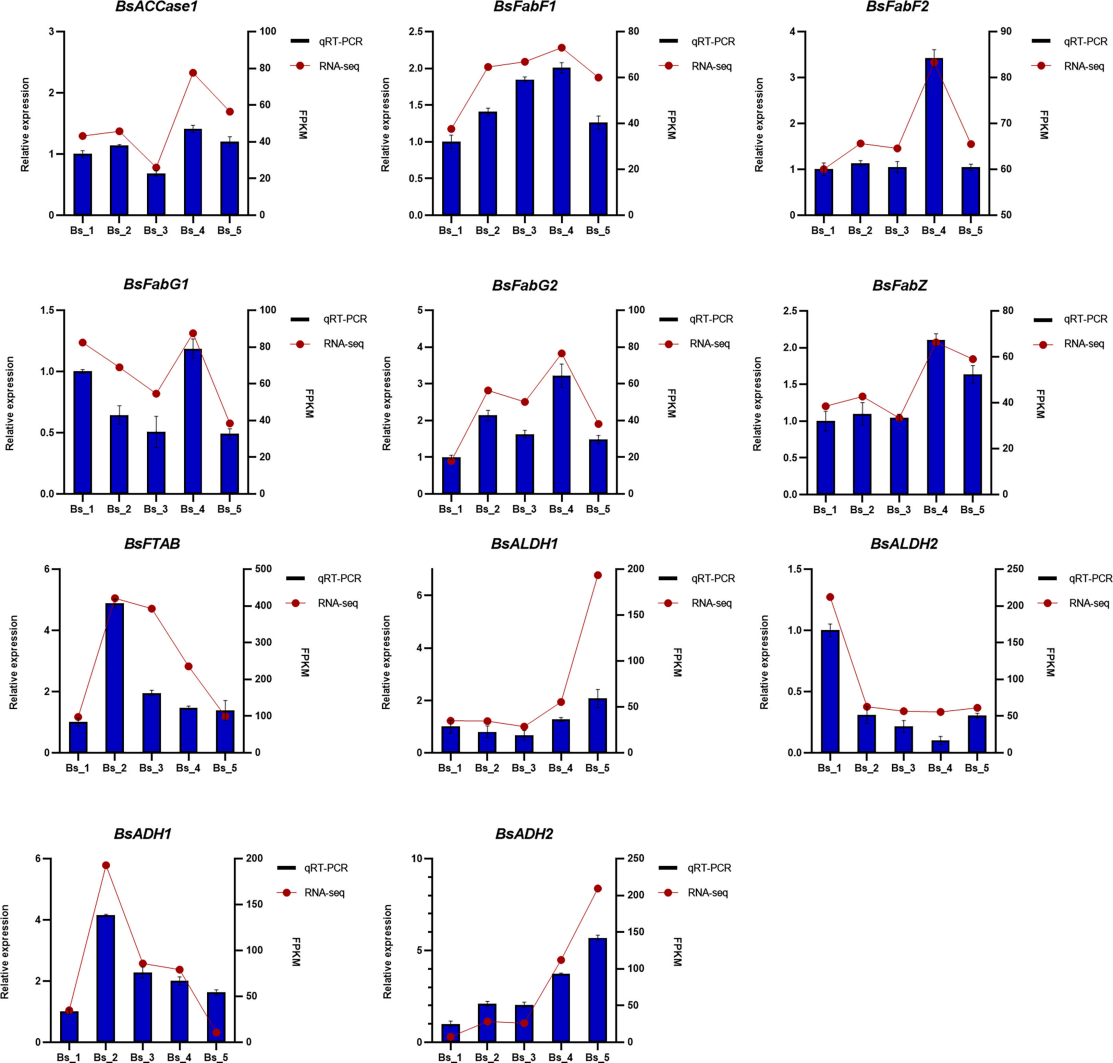
The qRT-PCR and FPKM-value analyses of 11 genes encoding enzymes involved in biosynthesis of fatty acids and their derivatives in *Bupleurum scorzonerifolium* root. Relative expression levels of 11 genes were analyzed, using the actin gene (*GAPDH*) as a reference for the normalization, all performed with three technical triplicates. Data represent mean ± SE.

To validate the reliability of the RNA-Seq data, 11 DEGs that may be involved in the biosynthesis of volatiles were selected for the qRT-PCR analysis. As shown in **Figure 9**, all the selected genes were differentially expressed at the five developmental stages, showing similar gene expression trends as the RNA-Seq results.

## 4 Discussion

*B. scorzonerifolium* root has been included in the Chinese, British and European Pharmacopoeias as an official medicine. BSVO is the essential chemical component of *B. scorzonerifolium* root, which is proven to possess various excellent biological activities, such as antibacterial, antipyretic, analgesic, anti-inflammation, and anti-depression. To reveal the changes in the composition and levels of volatile metabolites during the root development, their biosynthesis pathways were analyzed, and key regulatory genes were identified. Further, volatile metabolite profiling and transcript sequencing were performed.

### 4.1 Metabolic profiling of volatiles during the root development

The yield and quality of volatile oils from plants are closely related to the stage of plant development (Sousa et al., 2021). In the present work, BSVO continuously accumulated from the germinative to fruiting stages and slightly decreased from the fruiting to defoliating stages, indicating that the yield of BSVO generally increased with the root development and reached a maximum at the fruiting stage (**Figure 1**).

Previous studies reported that volatile oils from plants mainly composed of terpenes, fatty acids and their derivatives, and phenols (Schwab et al., 2008). Fatty acids and their derivatives were the characteristic volatile components of *B. scorzonerifolium* root. The contents of fatty acids and their derivatives accounted for 92.49 %-97.71 % of the total volatiles during the root development in *B. scorzonerifolium*, among which the contents of dodecanal, decyl acetate, 1-dodecanol, 1-decanol and dodecanoic acid were significantly higher than those of other compounds, indicating that they might be the main functional substances of BSVO (**Table S2**).

Among the 22 DAMs, 12 were classified as fatty acids and their derivatives (**Table S3**). The contents of dodecanal, decyl acetate, 1-dodecanol, decanal and tetradecanal were the maximum at the fruiting stage, whereas those of 1-decanol, dodecanoic acid, decanoic acid, hexadecanoic acid, undecanoic acid and 1-undecanol were the maximum at the vegetative stage. Additionally, the content of undecanal was higher at the florescence stage. Therefore, these DAMs could be used as chemical markers to characterize the changes in the levels of volatiles during *B. scorzonerifolium* root development.

In previous researches, 1-dodecanol, dodecanal and decanal were confirmed to have antibacterial, antiviral, tyrosinase inhibition, anxiolytic and sedative activities (Snipes et al., 1977; Davis-Hanna et al., 2007; Togashi et al., 2007; Hajhashemi et al., 2010; Hall et al., 2011; Mukherjee et al., 2013; Murray et al., 2019; Giovagnoni et al., 2022). These compounds were the dominant volatiles of BSVO and accumulated mainly at the fruiting stage. In addition, the yield of BSVO also peaked at the fruiting stage. Thus, the comprehensive analysis concluded that the optimal harvest time of *B. scorzonerifolium* root might be at the fruiting stage.

### 4.2 Molecular mechanism of biosynthesis of volatiles

Fatty acid metabolism is the most important pathway for the biosynthesis of fatty acids and their derivatives. The expression of *BsACCase1* and *BsACCase2* was highest at the vegetative and fruiting stages, respectively, and their upregulations probably promotes the production of malonyl-CoA, thereby providing abundant substrate for the downstream fatty acid synthase (FAS) complex (including FabF, FabG, FabZ, etc). Sequentially, the FAS system is responsible for channelling substrate from two-carbon acetate and acetyl-CoA to long-chain fatty acid. In this study, the DEGs included 6 genes encoding FAS, and the expressions of most of these genes were upregulated at the vegetative and fruiting stages, indicating that fatty acid biosynthesis was more active at these two stages. After the processing by FAS, the final step in the fatty acid biosynthesis pathway is the release of free fatty acid from fatty acyl-ACP, which is catalyzed by FAT (Bonaventure et al., 2003). In plants, FATA and FATB are the two different classes of FAT, with FATB preferentially acting on 8-18 carbon substrates (Jones et al., 1995; Voelker et al., 1997). Among these fatty acids detected in the volatiles, dodecanoic acid (C12 fatty acid) and decanoic acid (C10 fatty acid) were the most abundant, suggesting that *BsFATB* played a crucial role in regulating the fatty acid composition. During the root development, the transcript abundance of *BsFTAB* increased most significantly (4.3-fold) from the germination to vegetative stages and decreased from the vegetative to defoliating stages, indicating that there was more production of free fatty acids was observed at the vegetative stage (**Table S6**). Although ACCase and FAS were highly expressed at the fruiting stage, the expression of *BsFATB* was significantly decreased, resulting in lower content of fatty acids at the fruiting stage. These results were consistent with the findings of metabolomic analysis.

Unsaturated fatty acid is precursor for forming fatty aldehyde and fatty alcohol. In total, 27 genes, including 4 genes encoding FAB and 23 genes encoding FAD, were involved in desaturating fatty acyl-ACP to unsaturated fatty acids, which provided abundant precursor for further generation of fatty aldehydes and fatty alcohols. It has been clarified that C6/C9 aldehydes and alcohols are produced from α-linolenic acid *via* the LOX pathway (Ul Hassan et al., 2015). Two genes (*BsLOX1* and *BsLOX2*) may play a regulatory role in generating C6/C9 aldehydes and alcohols. Medium-chain fatty aldehydes, such as dodecanal (C12) and decanal (C10), were the major volatile compounds. However, unfortunately, their biosynthesis mechanism remains unknown. Recently, fatty acyl-CoA reductase (FAR) is reported to catalyze the formation of fatty aldehydes from fatty acyl CoA (Vioque and Kolattukudy, 1997; Doan et al., 2012). In *B. scorzonerifolium* root transcripts, five genes encoding FAR were annotated, however, their FPKM values were all less than 1. Therefore, it could not be determined whether the production of medium-chain fatty aldehydes were catalyzed by FAR. Notably, *BsFAB1* encoding FAB was responsible for desaturating hexadecanoyl-ACP to hexadecenoyl-ACP and octadecanoyl-ACP to octadecenoyl-ACP. The functional annotation information of *BsFAB1* in NR and Swiss-Prot databases is “palmitoyl-ACP 4-desaturase”. It has been shown that Δ^4^-palmitoyl-ACP desaturase catalyzes Δ^4^ desaturation of palmitoyl-ACP to produce petroselinic acid and Δ^4^-hexadecenoic acid (Cahoon and Ohlrogge, 1994). In addition, LOX and hydroperoxide lyase (HPL) were classified into dioxygenases and cytochrome P450 families, respectively, which together break the double bond at the C9 or C13 of α-linolenic acid to generate C6 or C9 aldehydes (Ul Hassan et al., 2015). In *B. scorzonerifolium* root, many genes encoding dioxygenases and cytochrome P450 were annotated. Therefore, it was speculated that the generation of medium-chain fatty aldehydes is *via* a similar pathway in *B. scorzonerifolium* root, such as oxidative cleavage of the double bond at C-4 of Δ^4^-hexadecenoic acid, resulting in dodecanal production. However, this speculation needs to be experimentally verified, the related enzymes should be further explored. Moreover, the biosynthesis pathway of medium-chain fatty aldehydes in *B. scorzonerifolium* root still needs to be further elucidated.

ALDH is the key enzyme responsible for the oxidation of aldehydes to carboxylic acids. In total, 24 ALDH families (ALDH1-24) were reported. Among them, ALDH2 and ALDH3 families are the most abundant in plants, with the former oxidizing mainly short-chain fatty aldehydes and the latter preferentially oxidizing medium- to long-chain fatty aldehydes (Stiti et al., 2021; Guan et al., 2022). Of the three genes encoding ALDH, *BsALDH1* and *BsALDH2* were classified as ALDH3, and *BsALDH3* was classified as ALDH2, which contributed to the conversion of short or medium-chain fatty aldehydes to the corresponding fatty acids. Interestingly, expressions of *BsALDH1* and *BsALDH2* showed opposite expression trends during the root development, suggesting that they respectively played roles in converting medium-chain fatty aldehydes to the corresponding fatty acids at the different developmental stages. ADH reversibly converts aldehyde to its corresponding alcohol (Dixon and Hewett, 2000). In *B. scorzonerifolium* root, *BsADH1*, *BsADH3* and *BsADH5* were upregulated at the vegetative stage, whereas *BsADH2* was sharply upregulated from the florescence to the defoliating stages. This suggested that they together promoted the biosynthesis of fatty alcohols during the root development.

Evidence suggests that many TF families, such as MYB, AP2/ERF, WRKY, TCP, GRAS and C2C2, are involved in the biosynthesis of fatty acids and their derivatives (Yang et al., 2022). In plants, WRKY is one of the largest families of TFs, and the family members of WRKY genes are reported to be involved in the production of some volatile compounds as regulators (Liu et al., 2017; Luo et al., 2020). Recently, WRKY6 was revealed to increase the content and change the composition of fatty acids in *Arabidopsis* (Song et al., 2020). In this study, three genes encoding WRKY6 were identified, and it is worth noting that one of them (*BsWRKY6_2*) presented the highest FPKM value at the vegetative stage, which was 13.88-fold more than that at the germinative stage. This result was consistent with the result that fatty acids mainly accumulated at the vegetative stage. There was evidence that MYB73 promoted the accumulation of fatty acids in *Arabidopsis* and *Lotus*, possibly through the downregulation of GLABRA2 expression (Liu et al., 2014). Here, three genes encoding MYB73 were detected to be involved in the production of fatty acids. According to reports, Dofs are plant-specific TFs that regulate various physiological processes in plants. (Yanagisawa, 2002; Le Hir and Bellini, 2013). Overexpression of soybean Dofs in *Arabidopsis* increased the content of fatty acids by activating the expression of ACCase and long-chain-acyl CoA synthetase gene (Wang et al., 2007). In this study, Dof4.6 and Dof11 genes were annotated from the DEMs, and they maybe promote the biosynthesis of fatty acids by regulating ACCase expression. Moreover, fatty acid biosynthesis is associated with phytohormone signalling. DELLA is a negative regulator involved in the gibberellins phytohormone signalling pathway (Chen et al., 2012; Yan et al., 2021). In this study, five genes encoding DELLA with FPKM values greater than 1 were detected, four of which were downregulated at the vegetative stage. This is consistent with the highest fatty acids content at this stage. In addition, RAP2.3, an AP2/ERF family TF, has been reported to improve the activity of ADH, which is responsible for fatty alcohol production (Pan et al., 2019). A gene encoding RAP2.3 was annotated in this work, and its transcript abundance was the highest at the fruiting stage, which is consistent with the result of fatty alcohols content. In summary, multiple TFs played positive or negative regulatory roles in the biosynthesis of fatty acids and their derivatives, leading to changes in the types and contents of fatty acids and their derivatives in *B. scorzonerifolium* root at the different developmental stages.

### 4.3 Correlation network analysis mining key genes

The correlation analysis between 12 DAMs and 65 related regulatory DEGs showed that 42 genes were significantly positively or negatively correlated with 12 DAMs. *BsFabF1*, *BsFAD4*, *BsFAD12*, *BsFAD13*, *BsFAD14*, *BsALDH2*, *BsMYB73_1*, *BsFAB2*, *BsFAD11*, *BsFAD20* and *BsDELLA5* were highly correlated with most DAMs, indicating that these genes had a more significant impact on the biosynthesis of fatty acids and their derivatives. Decanal and 1-decanol showed significant associations with most DEGs, followed by undecanal, dodecanal, 1-dodecanol and decyl acetate. Among 42 related DEGs, 23 genes encoded FAD, suggesting that fatty aldehydes, fatty alcohols, and fatty esters were probably formed from unsaturated fatty acids. Additionally, *BsFAB1* was highly correlated with dodecanal, 1-dodecanol and decyl acetate, further supporting the hypothesis that Δ^4^-hexadecenoic acid may be a precursor for dodecanal formation.

## 5 Conclusion

BSVO, a major chemical component of *B. scorzonerifolium* root, was continuously accumulated during *B. scorzonerifolium* root development and reached the maximum level at the fruiting stage. A total of 82 volatiles were detected from *B. scorzonerifolium* root, with fatty acids and their derivatives accounting for the largest proportion. Of these volatiles, 22 compounds were identified as DAMs, of which 12 were classified as fatty acids and their derivatives. Dodecanal, decyl acetate, 1-dodecanol, 1-decanol, decanal, tetradecanal, dodecanoic acid, decanoic acid, hexadecanoic acid, undecanoic acid, 1-undecanol and undecanal can be used as chemical markers to characterize the variation in the volatiles during *B. scorzonerifolium* root development. Furthermore, dodecanal, decyl acetate and 1-dodecanol were the dominant volatiles, which were mainly accumulated at the fruiting stage. Considering the yield of BSVO, the optimal harvest time of *B. scorzonerifolium* root may be at the fruiting stage. RNA-Seq and comparative analysis revealed a large number of DEGs during *B. scorzonerifolium* root development. 65 DEGs involved in the biosynthesis of volatiles were identified, and they encoded various enzymes and TFs, such as ACCase, FabF, FabG, FabZ, FATB, FAB, FAD, LOX, ALDH, ADH, TCP4, TCP20, MYB123, MYB73, WRKY6, WRKY43, DELLA, dof4.6, dof11 and RAP2.3. Of the 65 DEGs, 42 DEGs were significantly correlated with DAMs, indicating that these DEGs were the key genes regulating the biosynthesis of volatiles. Regulation of these key genes could promote the biosynthesis of fatty acids and their derivatives, improve the quality of BSVO, and enhance its clinical effect. This study provides important references for future studies to determine the harvest time of *B. scorzonerifolium* root and improve the quality of BSVO.

## Data availability statement

The datasets presented in this study can be found in online repositories. The names of the repository/repositories and accession number(s) can be found in the article/**Supplementary Material**.

## Author contributions

DY, WW and YC conceptualized and designed the experiment. DY and YC performed a study of the biosynthesis mechanism of volatile oils from *Bupleurum scorzonerifolium* Willd. root, analyzed the data and drafted the manuscript. WW conducted the analysis of volatile oils content and volatile metabolite profile. JH and YZ assisted in the analysis of the chemical constituent. XD supervised and edited the manuscript. All authors contributed to the article and approved the submitted version.
