# Supplementary material for: Study on molecular mechanism of volatiles variation during Bupleurum scorzonerifolium root development based on metabolome and transcriptome analysis
Source: Front Plant Sci. 2023 Mar 24;14:1159511. doi: 10.3389/fpls.2023.1159511 (PMC10079991; doi:10.3389/fpls.2023.1159511)
Supplement: Supplementary file 1 [file DataSheet_1.zip › SM file captions.docx]

Supplementary Material

**Study on** **molecular mechanism of volatiles** **variation during *Bupleurum scorzonerifolium* root development based on metabolome and transcriptome analysis**

**Dan Yu^1^, Wenxue Wang^1^, Jinhai Huo^2^, Yan Zhuang^2^, Yiyang Chen^1^, and Xiaowei Du^1*^**

*** Correspondence:**Corresponding Author: Xiaowei Du
E-mail: xiaoweidu@hotmail.com

# Supplementary Figures and Tables

## Supplementary Figures

**Supplementary Figure 1.** Principal component analysis of the fifteen samples based on the total transcripts. Samples labeled BS_1, BS_2, BS_3, BS_4, and BS_5 are the samples of *B. scorzonerifolium* root collected at germinative, vegetative, florescence, fruiting and defoliating stages, respectively.

**Supplementary Figure 2.** GO annotation analysis of the upregulated genes and downregulated genes in different compared groups. Samples labeled BS_1, BS_2, BS_3, BS_4, and BS_5 are the samples of *B. scorzonerifolium* root collected at germinative, vegetative, florescence, fruiting and defoliating stages, respectively.

**Supplementary Figure 3.** GO enriched analysis of DEGs in different compared groups. Samples labeled BS_1, BS_2, BS_3, BS_4, and BS_5 are the samples of *B. scorzonerifolium* root collected at germinative, vegetative, florescence, fruiting and defoliating stages, respectively.

**Supplementary Figure 4.** KEGG annotation analysis of DEGs in different compared groups. Samples labeled BS_1, BS_2, BS_3, BS_4, and BS_5 are the samples of *B. scorzonerifolium* root collected at germinative, vegetative, florescence, fruiting and defoliating stages, respectively.

**Supplementary Figure 5.** KEGG enriched analysis of DEGs in different compared groups. Samples labeled BS_1, BS_2, BS_3, BS_4, and BS_5 are the samples of *B. scorzonerifolium* root collected at germinative, vegetative, florescence, fruiting and defoliating stages, respectively.

## Supplementary Tables

**Supplementary Table 1.** The primers for qRT-PCR.

**Supplementary Table 2.** The content of volatiles.

**Supplementary Table 3.** Screening of DAMs.

**Supplementary Table 4.** The statistics of gene annotation success rate.

**Supplementary Table 5.** The number of DEGs in ten groups.

**Supplementary Table 6.** The FPKM of the selected genes.

**Supplementary Table 7.** Statistical table of transcription factor families.

**Supplementary Table 8.** Transcription factors involved in volatiles biosynthesis.

**Supplementary Table 9.** Correlation analysis of DEGs and DAMs.
