# Supplementary figures and images for: Study on molecular mechanism of volatiles variation during Bupleurum scorzonerifolium root development based on metabolome and transcriptome analysis
Source: Front Plant Sci. 2023 Mar 24;14:1159511. doi: 10.3389/fpls.2023.1159511 (PMC10079991; doi:10.3389/fpls.2023.1159511)

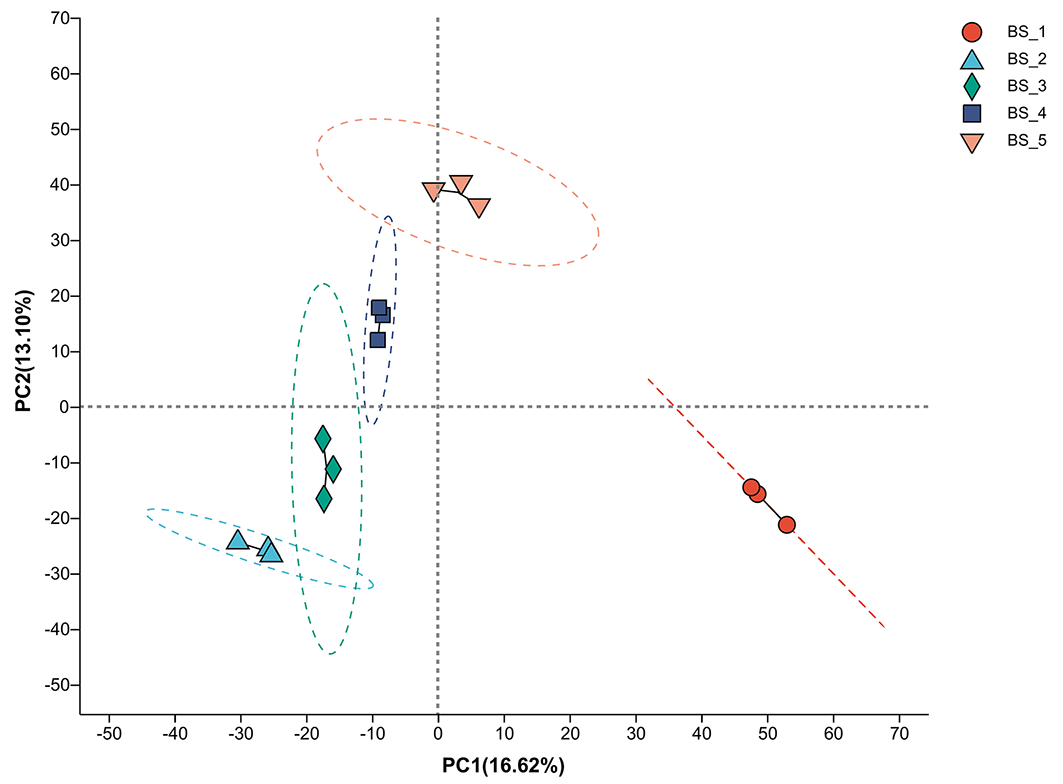

Supplement: Supplementary file 1 [file DataSheet_1.zip › Supplementary Material/Figure S1.tif]

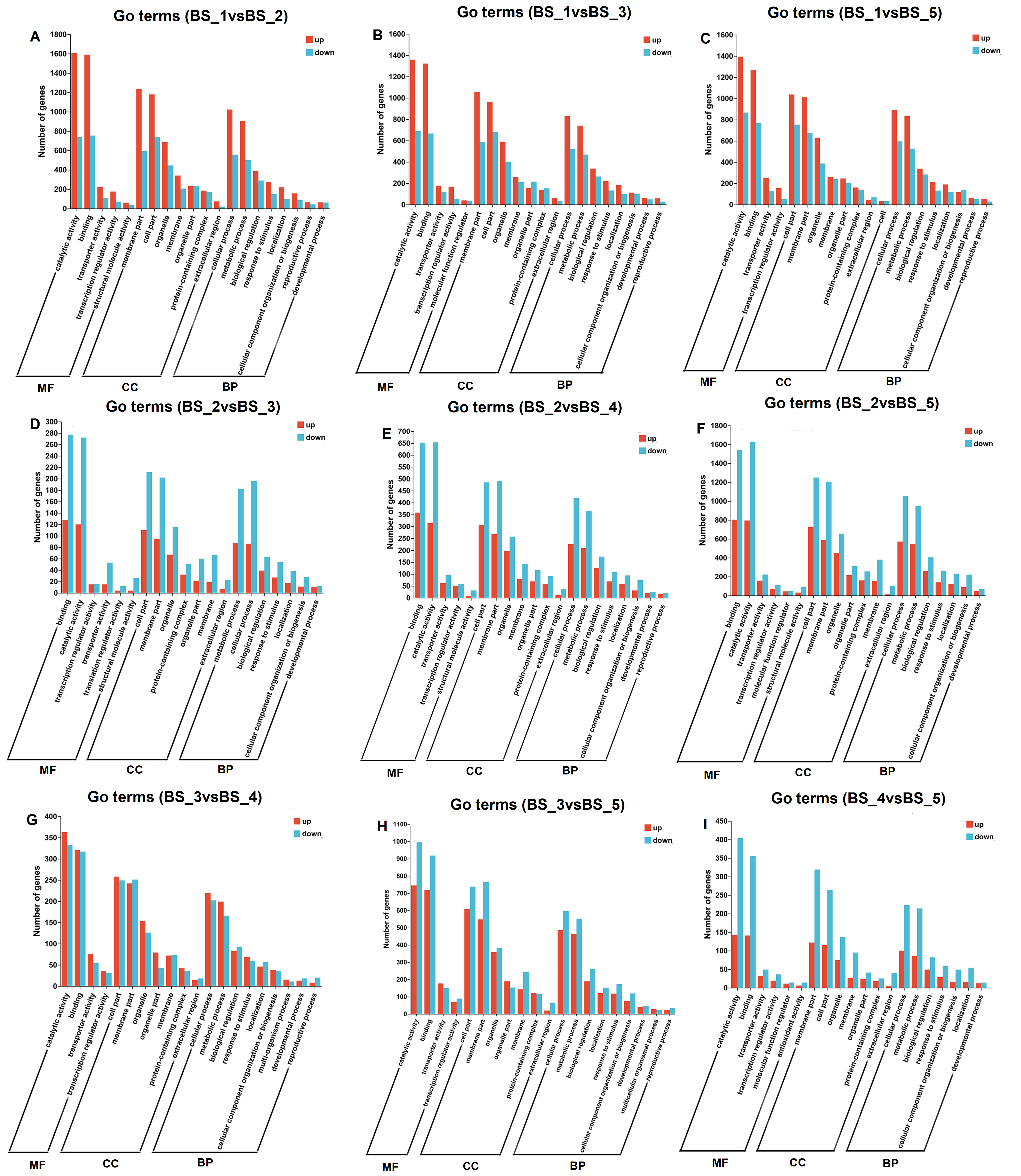

Supplement: Supplementary file 1 [file DataSheet_1.zip › Supplementary Material/Figure S2.tif]

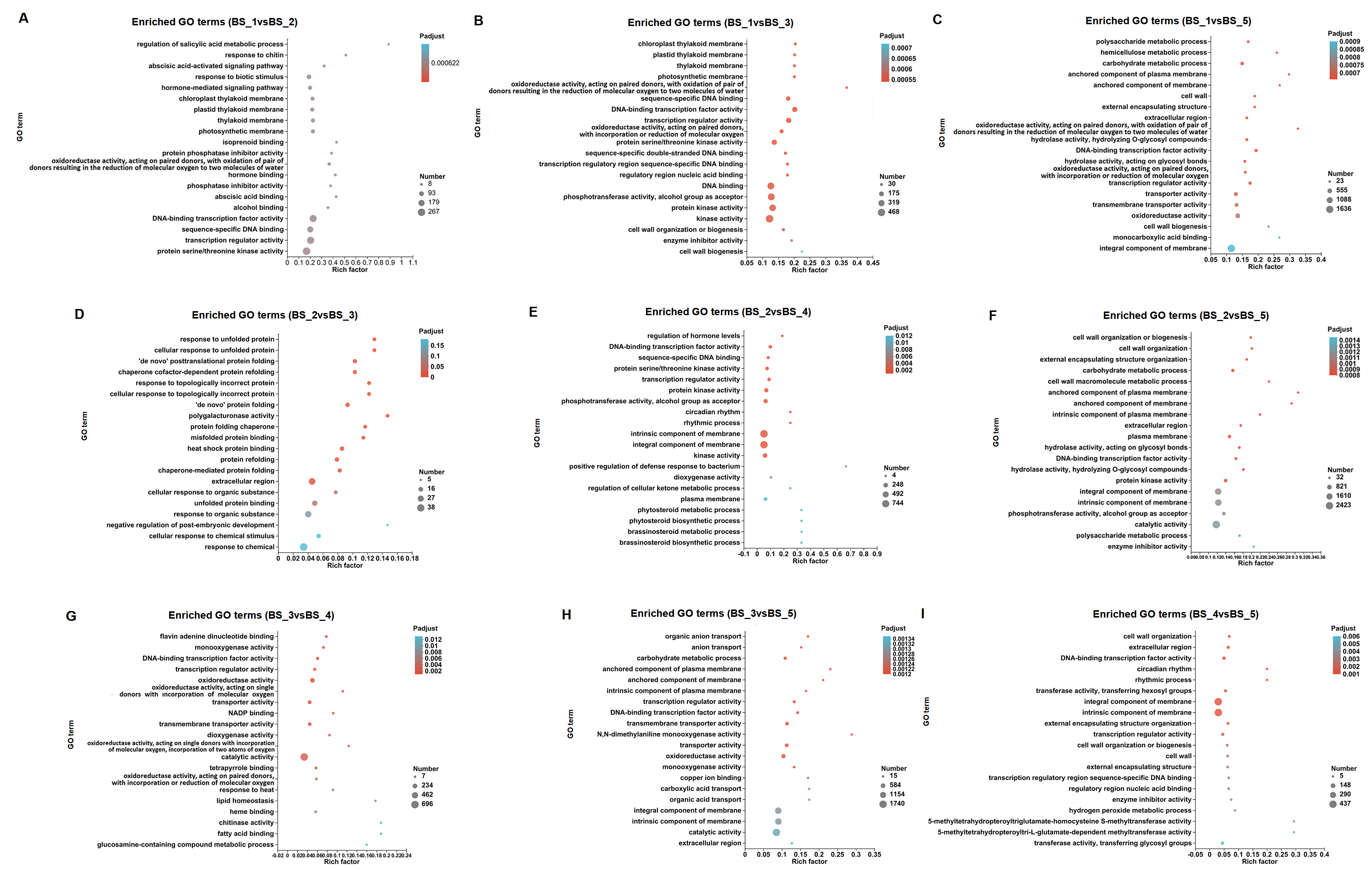

Supplement: Supplementary file 1 [file DataSheet_1.zip › Supplementary Material/Figure S3.tif]

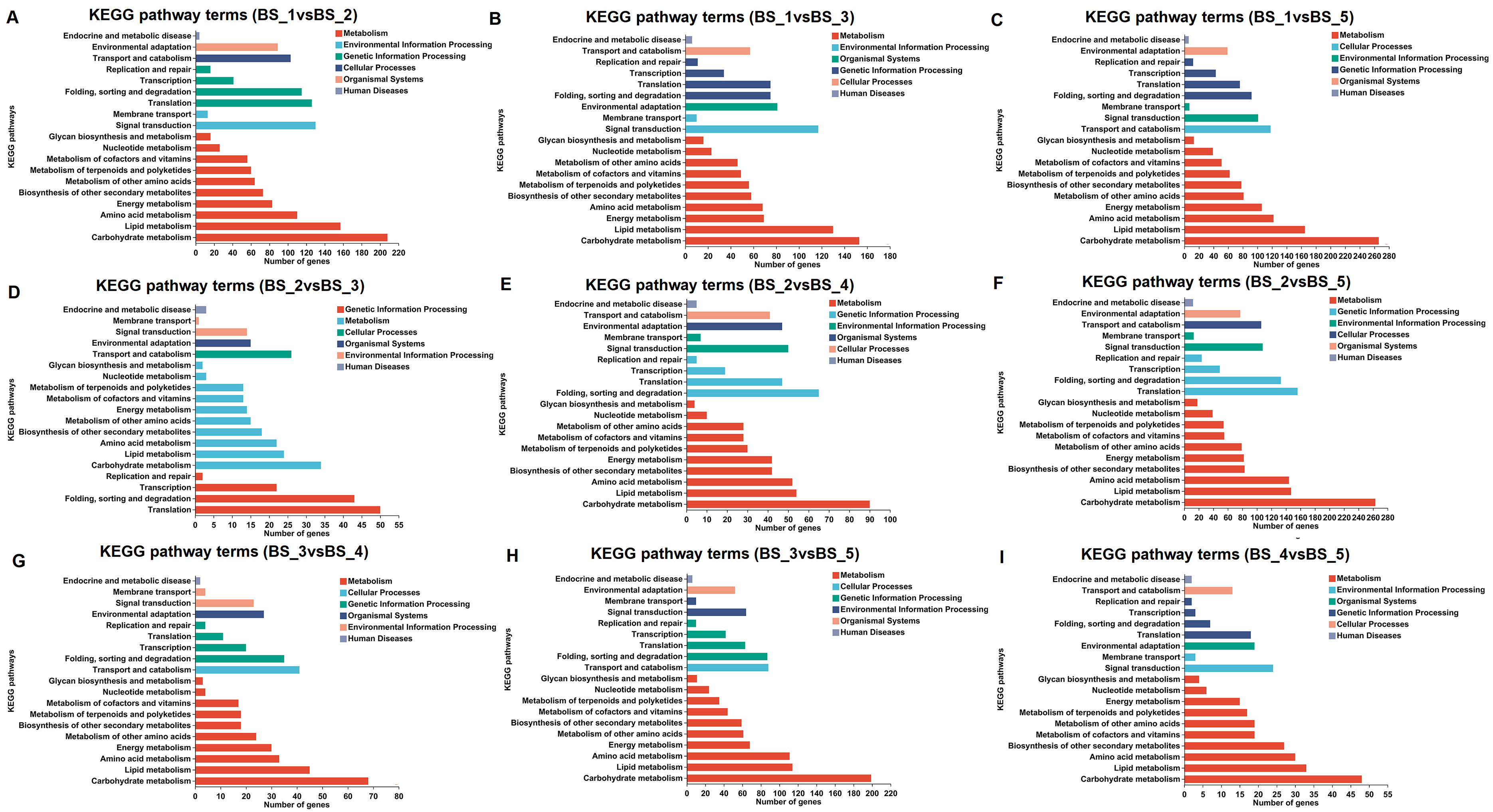

Supplement: Supplementary file 1 [file DataSheet_1.zip › Supplementary Material/Figure S4.tif]

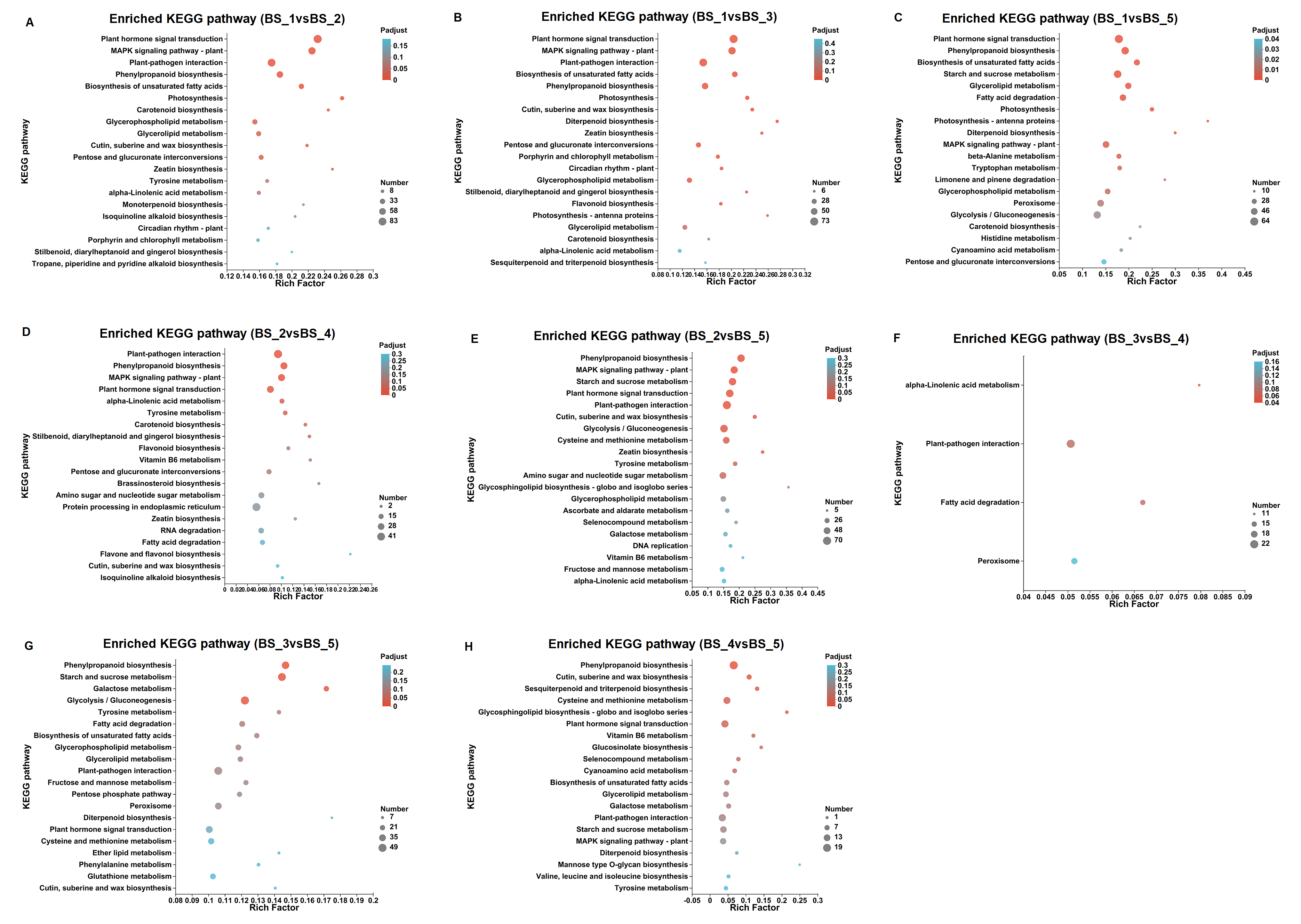

Supplement: Supplementary file 1 [file DataSheet_1.zip › Supplementary Material/Figure S5.tif]
